# Evaluation of MLVA for epidemiological typing and outbreak detection of ESBL-producing *Escherichia coli* in Sweden

**DOI:** 10.1186/s12866-016-0922-1

**Published:** 2017-01-06

**Authors:** Lisa Helldal, Nahid Karami, Christina Welinder-Olsson, Edward R. B. Moore, Christina Åhren

**Affiliations:** 1 Department of Infectious Diseases, Institution of Biomedicine, Sahlgrenska Academy and Centre for Antibiotic Resistance Research (CARe) at the University of Gothenburg, Gothenburg, Sweden; 2 Swedish strategic programme against antibiotic resistance, Region Västra Götaland, Gothenburg, Sweden; 3 Clinical Microbiology/Section for Bacteriology, Sahlgrenska University Hospital, Guldhedsgatan 10A, 413 46 Gothenburg, Sweden

**Keywords:** ESBL, *Escherichia coli*, O25b-ST131, H30-Rx ST131, Epidemiological typing, MLVA

## Abstract

**Background:**

To identify the spread of nosocomial infections and halt outbreak development caused by *Escherichia coli* that carry multiple antibiotic resistance factors, such as extended-spectrum beta-lactamases (ESBLs) and carbapenemases, is becoming demanding challenges due to the rapid global increase and constant and increasing influx of these bacteria from the community to the hospital setting. Our aim was to assess a reliable and rapid typing protocol for ESBL-*E. coli*, with the primary focus to screen for possible clonal relatedness between isolates. All clinical ESBL-*E. coli* isolates, collected from hospitals (*n* = 63) and the community (*n* = 41), within a single geographical region over a 6 months period, were included, as well as clinical isolates from a polyclonal outbreak (ST131, *n* = 9, and ST1444, *n* = 3). The sporadic cases represented 36 STs, of which eight STs dominated i.e. ST131 (*n* = 33 isolates), ST648 (*n* = 10), ST38 (*n* = 9), ST12 and 69 (each *n* = 4), ST 167, 405 and 372 (each *n* = 3). The efficacy of multiple-locus variable number tandem repeat analysis (MLVA) was evaluated using three, seven or ten loci, in comparison with that of pulsed-field gel electrophoresis (PFGE) and multi locus sequence typing (MLST).

**Results:**

MLVA detected 39, 55 and 60 distinct types, respectively, using three (GECM-3), seven (GECM-7) or ten (GECM-10) loci. For GECM-7 and −10, 26 STs included one type and eleven STs each included several types, the corresponding numbers for GECM-3 were 29 and 8. The highest numbers were seen for ST131 (7,7 and 8 types, respectively), ST38 (5,5,8) and ST648 (4,5,5). Good concordance was observed with PFGE and GECM-7 and −10, despite fewer types being identified with MLVA; 78 as compared to 55 and 60 types. The lower discriminatory power of MLVA was primarily seen within the O25b-ST131 lineage (*n* = 34) and its H30-Rx subclone (*n* = 21). Epidemiologically unrelated O25b-ST131 isolates were clustered with O25b-ST131 outbreak isolates by MLVA, whereas the ST1444 outbreak isolates were accurately distinguished from unrelated isolates.

**Conclusion:**

MLVA, even when using only three loci, represents an easy initial typing tool for epidemiological screening of ESBL-*E. coli*. For the ST131-O25b linage, complementary methods may be needed to obtain sufficient resolution.

## Background

Antibiotic resistance in pathogens is a growing problem in all parts of the world, making infectious diseases that have been curable for decades increasingly difficult to treat [1]. *Enterobacteriaceae* that harbour resistance mechanisms, such as extended-spectrum beta-lactamases (ESBL) and carbapenemases, pose major threats to human health [2]. *Enterobacteriaceae* harbouring ESBL were comparatively rare in Sweden and the other Scandinavian countries at the time of this study [3–6]. However, a few nosocomial outbreaks were reported [7–9]. Data from the European Centre for Disease Prevention and Control (ECDC) surveillance program show that the prevalence of *E. coli* with resistance to third generation cephalosporins is still low in the Scandinavian countries [10]. Further data have demonstrated that the prevalence with regard to ESBL-producing *Enterobacteriaceae* is low, although on the rise [11–14].

*Escherichia coli* Sequence Type 131 (ST131) has emerged as an important human pathogen and intestinal colonizer, having spread rapidly throughout the world and resulting in community and hospital-acquired urinary tract and bloodstream infections. *E. coli* ST131 is also considered to be a significant contributor in the increase in ESBL prevalence among infectious strains of *E. coli* [15, 16]. However, it has become evident that *E. coli* ST131 is not a single genotypic lineage, particularly demonstrable using pulsed-field gel electrophoresis (PFGE) for genomic profiling, but rather is an aggregate of distinctive sub-lineages. Its increasing antimicrobial resistance, including ESBL, has a strong cladistic base within the O25b:H4 serogroup and its *fimH30* lineage, with subsequent expansion to the H30R and H30-Rx subclones [2, 17, 18].

*Enterobacteriaceae* that harbour multiple resistance are widely distributed throughout the community, as part of the normal microbiota, even in areas of low prevalence of antibiotic resistance, such as in Scandinavia [11, 13]. Therefore, it is no longer feasible to implement surveillance measures for all isolates. Instead, the focus must be on identifying spread and timely alerts to halt outbreaks. For an outbreak situation that is epidemiologically evident, whole genome sequencing (WGS) is becoming the method of choice to confirm strain relatedness between isolates [19]. However, for standard consecutive infection control surveillance, there is a need for reliable typing systems that are easy and quick to handle with the primary aim to screen for clonal relatedness and often exclude rather than confirm strain identity. PFGE has been used extensively, but is becoming outdated due to several drawbacks, and, for the present purpose, is too slow and cumbersome. Several studies have explored the use of variable number of tandem repeat DNA sequences as a means of strain typing. Multiple-locus variable number of tandem repeat analysis (MLVA) is used to generate a DNA fingerprint of a bacterial isolate by PCR amplification and subsequent fragment analyses of target regions featuring Variable Numbers of Tandem Repeats (VNTR) distributed throughout the bacterial genome, and is increasingly being used for subtyping various species [20–22].

We have previously reported on the successful application of a generic *E. coli* MLVA (GECM) that uses 10 loci, as described by Lobersli et al. [23], to investigate a polyclonal plasmid-mediated ESBL-*E. coli* and *K. pneumoniae* outbreak [7, 24]. The GECM was shown to produce results that were comparable to those obtained with genomic profiling using PFGE. In most situations, isolates obtained during an outbreak will have an identical and unique typing profile. However, if the same profile is observed in isolates that are unrelated to that outbreak, information on the background distribution of the profile within the local bacterial population and a reasonable timeframe, becomes critical [25]. With the aim of deriving a typing method for ESBL-*E. coli* that can be used for standard consecutive infection control surveillance, we evaluated the efficacy of MLVA, using different numbers of loci, and compared it with PFGE and multi locus sequence typing (MLST), to distinguish consecutively identified ESBL-*E. coli* isolates, including the above mentioned outbreak isolates, in a municipal area in south-western Sweden whilst this longstanding, initially undetected, outbreak lasted.

## Methods

### Bacterial isolates

All of the ESBL-*E. coli* isolates detected at the Clinical Bacteriology Laboratory, Sahlgrenska University Hospital since 2003 are routinely stored at −70 °C and the epidemiological data linked to the isolates are documented. A previously described polyclonal, plasmid-mediated outbreak caused by ESBL-producing *E.coli* of two STs as well as *K. pneumoniae*, took place from September 2008 until it finally was revealed and terminated in December 2008 [7, 24]. For this study, almost all clinical, non-duplicate ESBL-*E. coli* isolates detected during the ongoing outbreak period and through the follow-up period, i.e. until February 2009, were included. Thus, all sporadic cases that could be retrieved (104/108) for further analyses were included. In addition, all clinical ESBL-*E. coli* samples, i.e. 12 isolates from seven patients, that were part of the above-mentioned outbreak were included. No additional outbreaks occurred during the study period, according to standard consecutive epidemiological surveillance performed at the time. The sporadic cases included isolates collected both from the hospital (*n* = 63) as well as from the community setting (*n* = 41). They corresponded to 36 STs, of which ST131 was the most common (*n* = 42). For 21 STs, only a single isolate was detected. For the sporadic cases, no ST dominated in a particular setting. The *E. coli* outbreak isolates belonged to ST131 (*n* = 9) and ST1444 (*n* = 3).

The *E. coli* isolates were identified according to the routine protocol at the laboratory, using conventional biochemical tests. Antibiotic susceptibility was determined, using the disc diffusion assay and breakpoints in practice at the time, according to the Swedish Reference Group for Antibiotics (SRGA) and in accordance with the recommendations of the European Committee on Antimicrobial Susceptibility Testing (EUCAST) [26]. Cephalosporin-resistant isolates were screened for the ESBL phenotype, using the double-disc diffusion assay [27]. For this study, frozen samples were retrieved and plated on blood agar medium. Two to five bacterial colonies from overnight cultures were suspended in 100 μl EDTA, heated for 15 min at 95 °C, and centrifuged at 18,000 × *g* for 5 min. Cell supernatants, containing the bacterial DNA, were separated from pelleted cell debris and used for the subsequent DNA analyses.

### Genotypic detection of bla_CTX-M_, bla_TEM_, bla_OXA_, bla_SHV_

All *E. coli* isolates that were characterized phenotypically as being ESBL-positive were confirmed, using a multiplex PCR assay for detecting the genes for the CTX-M-, TEM-, OXA- and SHV-β*-*lactamases, as previously described [28]. Subsequently, the isolates were investigated for CTX-M phylogroups, using a standardised PCR assay and a Taq Man PCR protocol [29].

### Pulsed-field gel electrophoresis

PFGE was performed, following the extraction of genomic DNA and digestion with restriction enzyme *Xba*I, as described previously by Welinder-Olsson et al. [30]. Briefly, *XbaI* digested DNA was electrophoresed in 1% agarose in 0.5 × TBE at 14 °C for 26 h, using the Gene Path system (Bio-Rad Laboratories, Sweden), set at 6 V/cm, with pulse times linearly increasing from 12.6 s initial switch time to 40 s final switch time. *Xba*I-digested DNA from *Salmonella* serotype Braenderup H9812 was included as a normalisation standard on every gel. The DNA band patterns were analysed, using the BioNumerics software version 6.6 (Applied Maths NV, Sint-Martens-Latem, Belgium) with the Dice coefficient for calculating pair-wise similarities, and the UPGMA algorithm for constructing dendrograms of estimated relatedness. Position tolerance and optimisation were set at 1.0. Bands within the size range 453-78 kb were included in the analysis. Interpretation of strain similarity was performed according to previously published guidelines [25, 31, 32]. For the present study, strains were assigned the same PFGE-type if the similarity was ≥80%. In addition to using the BioNumerics software for analysing band pattern similarity, visual interpretation of band differences was performed to ascertain the results. As generally recommended, isolates with as many as three band differences were then considered the same PFGE-type, corresponding to ≥80% similarity. The visual analyses of band patterns did not alter the results from using similarity index. The PFGE profile types were arbitrarily designated using a letter combination, the designated letters not indicating any similarity of pattern nor subtypes. For comparison between MLVA and PFGE results, PFGE types were also defined by using ≥70% similarity and ≥90% similarity.

### Multi-Locus Sequence Typing (MLST)

MLST was performed according to the method of the *E. coli* MLST database website [33] as previously described [7]. Representative isolates of all MLVA-types irrespective of number of loci used, as well as all PFGE-types, were sequenced. The sequences were submitted to the MLST database for *E. coli* and the respective sequence types were determined.

### Identification of *E. coli *O25b- ST131 and its H30-Rx subclone

Detection of the O25b-ST131 strain-type was performed, using an O25b-ST131 allele-specific *pabB* PCR [34]. The *adk*-gene was used as the positive control for the PCR assessing DNA quality, as described previously [7]. The H30 and H30-Rx subclones were detected, as described previously [17, 35].

### Generic *E. coli* MLVA (GECM)

GECM was performed targeting seven tandem sequence repeats (GECM-7; CVN001, CVN002, CVN003, CVN004, CVN007, CVN014, CVN015) initially described by Lindstedt et al., [36]. In addition, CVN016 and CVN017 and one regularly interspersed short palindromic repeat (CCR001), that have been described to increase the discriminatory power of this MLVA, were included (GECM-10) [23].

Four PCR assays with HotStarTaq Master Mix (Qiagen, Hilden, Germany) and multiple dye-labelled amplification primers were used, as described previously [23, 36]. The first PCR contained primers for the CVN003 and CVN014 loci, the second PCR had primers for CVN001, CVN004, CVN007 and CVN015, the third PCR contained primers for CCR001, CVN016 and CVN017, and the fourth PCR was a single PCR for the CVN002 locus. The final concentrations of the PCR primers were slightly modified, as described previously [7, 23, 36]. After the PCR amplifications, the first and fourth multiplex PCR products were pooled. To this pooled solution, 0.5 μl of the Geneflo-625 TAMRA (CHIMERx) internal size standard was added, with 12 μl of formamide, and subsequently electrophoresed, using the ABI-310 Genetic Analyzer (Applied Biosystems, Foster City, CA, USA), as described previously [36]. The second multiplex PCR product was handled in the same manner, whereas the third PCR product was mixed with Genescan-Liz600 (Applied Biosystems) instead of Geneflo-625 TAMRA. For all the loci, all of the individual peaks were identified, based on the dye-labelling and size. In addition to the length of each locus (allowing for ±1.5 base pairs difference), the numbers of repeats were calculated according to Lobersli et al. [23]. If no PCR-product was detected, the locus was denoted as ʻNʼ and if no repeats could be calculated, it was denoted ʻ0ʼ. All the GECM profile types were arbitrarily designated.

### Comparison of MLVA and PFGE results

The partitions comparison computer software was used for the assessment of diversity (Simpson’s index of diversity) [37].

## Results

### Overall strain typing results

Of the 116 isolates included, nine O25b-ST131 isolates and three ST1441 isolates were linked to the previously described polyclonal outbreak [7, 24], while the remaining isolates had no known epidemiological relationship and thus were considered sporadic cases. The sporadic cases represented 36 STs of which eight STs dominated i.e. ST 131 (*n* = 33 isolates), ST648 (*n* = 10), ST38 (*n* = 9), ST12 and 69 (each *n* = 4), ST 167, 405, and 372 (each *n* = 3). Two isolates were found in each of ST 10, 43, 58, 393, 443 and 1284 and the remaining STs were represented by only one isolate each.

**Fig. 1:**
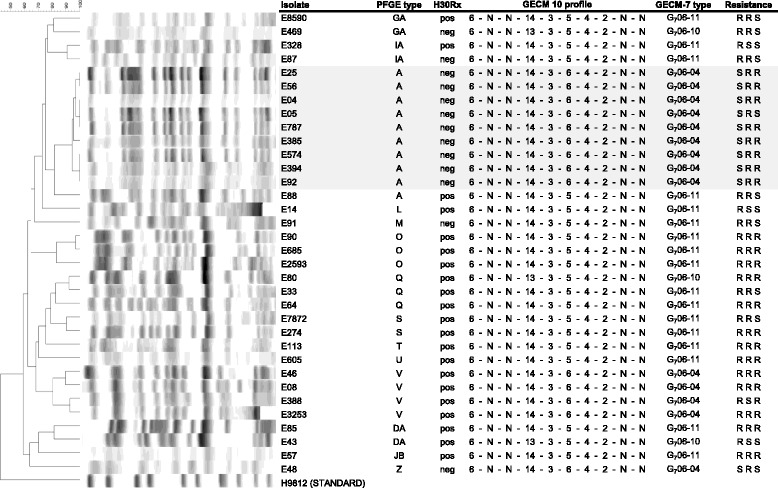
PFGE profile in relation to MLVA profile for isolates belonging to the O25b-ST131 linage. PFGE profile in relation to MLVA profile for 34 ESBL-*E. coli* isolates, including both sporadic cases and the outbreak isolates (shaded in grey) belonging to the O25b-ST131 lineage and its subclone H30Rx (pos). Names of PFGE-type using > 80% similarity index are given. The GECM profile corresponds to the number of repeats in the following loci; CVN 001, CVN 002, CVN 003, CVN 004, CVN 007, CVN 014, CVN 015, CCR 001, CVN 016 and CVN 017. N denotes no PCR-product and 0 denotes that no repeats could be calculated. The GECM-7 types are given, which correspond to MLVA using the first seven loci of GECM-10. Resistance (R) or sensitivity (S) for ciprofloxacin, trimethoprim and tobramycin

All isolates were typeable by generic *E. coli* MLVA. When the initial seven loci were analysed (GECM-7), 55 strain types were identified that differed by at least one allele; 38 of these were singletons, the remainder contained two to six isolates each, with the exception of two large clusters of isolates that were assigned to the O25b-ST131 lineage (Tables 1 and 2). Isolates belonging to the O25b-ST131 lineage (*n* = 34) and its subclones H30 (*n* = 33) and H30-Rx (*n* = 21) were clearly distinguishable from the other isolates and were found within three GECM-7 types (Fig. 1 and Table 1).

**Table 1:** Correlation between generic *E. coli* MLVA using seven loci (GECM-7) and PFGE using >80% similarity index for the designation of types for typing 116 ESBL-*E. coli* isolates; 34 isolates (bold) belong to the O25b-ST131 lineage

PFGE-types	GECM-7 types																		
	G_7_ 05-07	G_7_ 05-09	G_7_ 05-04	G_7_ 05-05	G_7_ 05-06	G_7_ 05-08	G_7_ 06-14	G_7_ 06-13	G_7_ 06-12	G_7_ 06-10	G_7_ 06-11	G_7_ 06-04	G_7_ 06-07	G_7_ 06-08	G_7_ 06-09	G_7_ 07-02	G_7_ 07-03	Singleton by GECM-7 and cluster by PFGE	Total
JB											**1** ^**b**^			2					3
CK													3						3
DA										**1** ^**b**^	**1** ^**b**^								2
GA										**1**	**1** ^**b**^								2
IA											**2** ^**b**^								2
A											**1** ^**b**^	**9** ^**a**^							10
O											**3** ^**b**^								3
Q										**1** ^**b**^	**2** ^**b**^								3
S											**2** ^**b**^								2
V												**4** ^**b**^							4
BU								3											3
BF					2														2
AR							2												2
AS							1											1	2
B																3^a^			3
CB				3															3
CG		2																	2
Non-typeable		1				1											1		3
Singleton by PFGE and cluster by GECM-7	2	1	2	1		1	2	3	2		**4** ^**b**^	**1**	1	1	2		1	Singleton PFGE and GECM-7 38^c^	Singleton PFGE 62^c^
Total	2	4	2	4	2	2	5	6	2	**3**	**17**	**14**	4	3	2	3	2	Singleton GECM-7 39	Total 116

^a^Isolates belonging to a polyclonal outbreak. ^b^Includes isolates belonging to the H30-Rx subclone (*n* = 21). ^c^Includes one isolate non-typeable by PFGE

**Table 2:** Comparison of the numbers of types generated by the various MLVA and PFGE. The numbers of types generated by the various MLVA using three (GECM-3), seven (GECM-7), or ten (GECM-10) loci, respectively, in comparison with that of PFGE using >80% similarity index for the designation of types, for the typing of 116 ESBL-*E. coli* isolates belonging to either the ST131-O25b linage (*n* = 34) or not

Number of isolates within a type	GECM-3		GECM-7		GECM-10		PFGE	
	Non-O25b	O25b	Non-O25b	O25b	Non-O25b	O25b	Non-O25b	O25b
Singleton	22	0	38	0	44	0	60^a^	6^b^
2–4	11	1	11	1	11	1	9^b^	7
5–9	2	0	3	0	2	0	0	0
≥10	1	2	0	2	0	2	0	1
Total number of types	36	3	52	3	57	3	69	14
Simpsons index of diversity (CI 95%)	0.96 (0.94–0.98)	0.59 -	0.98 (0.97–0.99)	0.59 -	0.99 (0.98–0.99)	0.59 -	1.0 (0.99–1.0)	0.89 -

^a^Isolates non typeable by PFGE (*n* = 4) included, each with an individual designation and thus accounted for as singeltons. ^b^PFGE type JB is included in both the non-O25b and O25b isolates (however both ST131), so it is listed in both columns

Four of the 116 isolates could not be typed using PFGE. Using a cut-off of ≥80% similarity for designation of PFGE types and excluding nontypable isolates, 78 types were identified, of which 61 were singletons (Tables 1 and 2). The remaining 17 clusters consisted of two to four isolates, apart from one larger cluster that included outbreak isolates (PFGE type A). The O25b-ST131 lineage and the H30-Rx subclone were detected within 14 and 12 different PFGE types, respectively. These clusters included one to four isolates each, with the exception of one of the outbreak clusters.

**Fig. 2:**
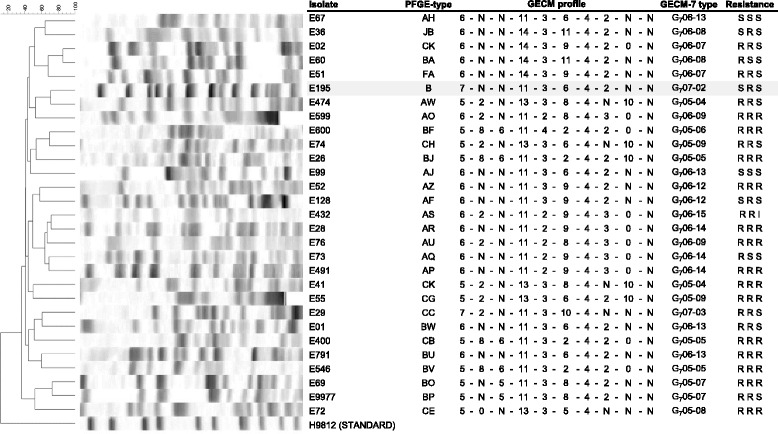
PFGE profile in relation to MLVA profile for isolates, not part of the O25b-ST131 lineage. PFGE profile in relation to MLVA profile for 29 ESBL-*E. coli* isolates, each representing PFGE types with several isolates and not part of the O25b-ST131 lineage. All are sporadic cases with the exception of one isolate shaded in grey that represents three outbreak isolates. Names of PFGE-type using > 80% similarity index are given. The GECM profile corresponds to the number of repeats in the following loci; CVN 001, CVN 002, CVN 003, CVN 004, CVN 007, CVN 014, CVN 015, CCR 001, CVN 016 and CVN 017. The GECM-7 types are given which corresponds to MLVA using the first seven loci of GECM-10. Resistance (R) or sensitivity (S) for ciprofloxacin, trimethoprim and tobramycin

Most of the sporadic isolates were resistant to ciprofloxacin (82%, 85/104), trimethoprim (79%, 71/104) or both (66%, 69/104). Resistance to tobramycin was less frequent (39%, 41/104), as was resistance to all three antibiotics (36%, 37/104). The corresponding resistance rates for the nine ST131-O25b outbreak isolates were; tobramycin 8/9, trimethoprim 9/9 and ciprofloxacin 0/9, and for the three ST1444 outbreak isolates 2/3, 3/3, and 3/3, respectively. The antibiograms showed no coherence with GECM-7 types or PFGE-types (Figs. 1 and 2). The vast majority of the isolates carried ESBLs of the CTX-M-1 (90/116) or CTX-M-9 (20/116) phylogroup.

### Comparison of MLVA and MLST

Representative isolates from each of the MLVA-types were analysed by *E. coli* MLST. Among the 37 STs detected, 26 STs included only one MLVA-type determined by using 7 loci (GECM-7), each with one to three isolates/type. The remaining 11 STs included several, i.e. two to seven, GECM-7 types within each ST, the highest numbers of types seen for ST648 (5 types, 10 isolates), ST38 (5 types and 9 isolates) and ST69 (4 types, 4 isolates) in addition to ST131. The latter included 42 isolates separated into seven GECM-7 types, of which the O25b-ST131 lineage corresponded to three types (34 isolates). The nine O25b negative isolates of ST131 were found within four types with one to three isolates/type.

Using 10 loci (GECM-10) resulted in almost identical results as those of GECM-7, i.e. 26 STs included only one GECM-10 type and 11 STs several types. Only two STs were further subdivided into additional types, i. e. ST131 and ST38, by adding the three extra loci of GECM-10 to those of the GECM-7 protocol. Using only three loci (GECM-3), 29 ST included only one MLVA-type, and eight STs each included two to seven types, with one less type in ST405, 648, 617 and 1284, as compared to using seven or ten loci.

Altogether, the highest numbers of MLVA-types were seen for ST131 (7,7 and 8 types for 3,7 and 10 loci, respectively), followed by ST38 (5,5,8 types), ST648 (4,5,5 types), ST69 (4,4,4 types), ST12 (3,3,3 types), ST 10, 58, 443, (2,2,2 types), and ST 405, 617, 1284 (1,2,2 types).

### Comparison of MLVA (GECM-7) and PFGE for strain typing

The correlation, between GECM-7 and PFGE was good, as demonstrated in Table 1, when using a threshold of ≥80% similarity for the designation of PFGE types, even though GECM-7 exhibited a lower discriminatory capacity than PFGE, especially for the O25b-ST131 isolates (Tables 1 and 2). The PFGE-similarity index was in the range of 41–67% within a given GECM-7 type, except for those cases with complete concordance. Neither use of ≥70% nor ≥90% similarity for designation of PFGE types changed the level of concordance with MLVA.

The number of singletons detected by PFGE were almost twice as many as determined by GECM-7. Also, all of the PFGE non-typeable isolates were typeable by GECM-7. In the cases where a GECM-7 type included isolates belonging to several PFGE types a few isolates of two to three PFGE-singleton types generally were clustered into one GECM-7 type, or they were added to a PFGE cluster of two to three isolates, e.g. G_7_06-13 including 3 PFGE type BA isolates and 3 isolates of PFGE singleton types (Table 1). For the O25b-ST131, as well as for the H30-Rx isolates, discordance was evident especially within the G_7_06-11 type, which constituted 12 different PFGE types (Table 1).

In six instances, more than one GECM-7 type was found within a PFGE ≥80% similarity cluster (Table 1 and Figs. 1 and 2). In cases of type Q and AS, the similarity index for the isolates of different MLVA-types was 92 and 96%, respectively, whereas in the remaining four cases (i.e. PFGE types JB, DA, GA and A), the isolates were indeed not completely identical with PFGE profiling. When using the higher cut-off of ≥90% similarity, these isolates were separated into PFGE subtypes with 80–89% identity. Thus, in all of these cases, GECM-7 had a higher discriminatory power than PFGE, using ≥80% profile similarity for designation of types. This also included O25b negative ST131 isolates that could be distinguished from a O25b positive ST131 isolate, by GECM-7 but not by PFGE (type JB, Tables 1 and 2).

The ST1444 outbreak isolates were clustered and clearly distinguished from the epidemiologically unrelated isolates by both PFGE (type B) and GECM-7 (G_7_07-02). For the O25b-ST131 outbreak isolates, one epidemiologically unrelated isolate was added to the PFGE type A outbreak cluster (Table 1, Fig. 1). Using ≥90% profile similarity, as generally recommended for outbreak isolate identification, this isolate no longer clustered with that part of the outbreak. GECM-7 was able to separate this epidemiologically unrelated strain, although it was unable to separate other unrelated O25b-ST131 isolates from the outbreak isolates, including four isolates that clustered in a clearly distinct PFGE type (V).

### Alternative methods for generating MLVA data

In order to increase the discriminatory power of MLVA, Lobersli et al. added three loci, CCR001, CVN016 and CVN017, to the original seven loci included in GECM-7 [23]. Additional discriminatory power was achieved by adding the first two of these loci, with the exception of the O25b-ST131 isolates (Tables 2 and 3). This resulted in 60 distinct GECM-10 types, of which 44 were singletons. However, the GECM-10 did not elucidate the discrepancies observed between MLVA and PFGE. The most prominent changes that resulted from the use of GECM-10 instead of GECM-7 were noted for the four ST38 isolates within the GECM-7 type, G_7_05-09, all of which became singletons, as described above.

**Table 3:** Frequency (%) of isolates with the designated number of repeats within each locus

No of repeats^a^	CVN001		CVN002		CVN003		CVN004		CVN007		CVN014		CVN015		CCR001		CVN016		CVN017	
1															1					
2			29						13		15				61	**100**				
3									83	**100**	2		1		16		1			
4	1								4		5		96	**100**						
5	40				4						13	**59**			1					
6	40	**100**			10						20	**41**								
7	13				1						5		2				1			
8	5		10								11									
9							1				17									
10							2				6						7			
11							70				5						1			
12			1				2				1									
13							15	**9**												
14			1				8	**91**												
19			1																	
0			4														30			
N			54	**100**	85	**100**									17		59	**100**	100	**100**

^a^The outbreak isolates are included, i.e., nine O25b-ST131 isolates (GCEM-10 profile: 6-N-N-14-3-6-4-2-N-N) and three ST1444 isolates (GCEM-10 profile: 7-N-N-11-3-5-4-2-N-N)

Frequency (%) of isolates with the designated number of repeats within each of the loci for MLVA for the typing of 116 ESBL*-E. coli* isolates. The bolded loci correspond to GECM-3 type MLVA and the seven first loci to that of GECM-7. The bold frequencies represent isolates that belong to the O25b-ST131 lineage (*n* = 34)

We also evaluated the use of either a system of arbitrary designations assigned to each allele, as first suggested by Lindstedt et al. [36], or the actual fragment size for designation of MLVA types as used by others [22, 38], but we found very minor deviations from our results obtained using repeat numbers.

### Potential of MLVA for typing in standard consecutive infection control surveillance

The number of repeats obtained within each loci varied greatly (Table 3). Locus CVN017 was not detected at all, and neither were CVN002, CVN003, and CVN016 in 78, 104, and 81 of the studied isolates, respectively. The largest variation was seen for CVN014, followed by CVN001 and CVN004. For the O25b-ST131 lineage and its H30-Rx subclone, variation within each loci was comparatively low; four loci were absent, four were monomorphic and the remaining two (CVN014 and CVN004) were dimorphic with regard to the number of detected repeats/loci, Nor could these lineages be distinguished from the other isolates based on the presence of a distinct allele within a certain locus.

Using the three loci of CVN001, CVN004, and CVN014, which were present in all the isolates and showed variations in the number of repeats, a mini-MLVA, here designated as ʻGECM-3ʼ, was evaluated for application to the initial screening of suspected transmission based on standard consecutive epidemiological screening. The discriminatory power of GECM-3 was not much lower than that of GECM-7 or GECM-10. Even though additional clustering was noted, only three clusters with ≥5 isolates were formed (Table 2). The ST1444 outbreak isolates could still be distinguished from the other isolates. As expected, the results for the O25b-ST131 lineage were unaltered when GCEM-3 was used.

## Discussion

The rapidly increasing number of ESBL-*E.coli* isolates and the continuous influx of strains from the community to hospital settings [4, 15, 39], make the surveillance of hospital transmission and alerting to potential outbreaks a demanding challenge, even in low endemic settings. With the aim of addressing this issue, we evaluated the use of a generic *E. coli* MLVA [23, 36] on a set of clinical isolates, representing more than 37 different STs and 78 PFGE-types. The isolates represented almost all consecutive sporadic cases from an entire region, over a period of 6 months while a longstanding, initially undetected polyclonal outbreak was ongoing [7]. The sporadic cases had no apparent epidemiological relationship with this outbreak nor with each other. Despite this, but not surprisingly, clustering of isolates occurred in varying degrees, depending on the method used, most evident for MLST, followed by MLVA using three loci. PFGE was the most discriminatory method, especially with regard to the widespread ST131-O25b clone. Outbreak isolates belonging to this linage were clustered with other sporadic cases using all methods; only PFGE using ≥90% similarity index clearly distinguished these outbreak isolates from the sporadic cases. The ST 1441 outbreak isolates were separated from the sporadic cases with all methods tested.

MLVA that employed seven or ten loci showed almost equivalent performance levels, and since the use of ten loci for the MLVA (GECM-10) only slightly increased the number of newly detected MLVA types, and only within a limited number of STs, we focused on evaluating the performance of the protocol with seven loci (GECM-7). However, some diversity in terms of the numbers of repeats detected for the isolates was seen in two of the three additional loci of the GCEM-10. The addition of one of these loci improved the discriminatory capacity, as reported in our previous study [7]. Similar findings have been reported by others [40], suggesting that these loci could be of interest to add when the use of fewer loci generates inconclusive results.

The MLVA types corresponded to almost half the number of STs. A subset of 11 STs were subdivided into several MLVA-types, most prominent for ST38, ST648 and the ST131 cluster, when including isolates both within and without the ST131-O25b lineage. By increasing the number of loci from three to seven, ST 405, 617, 648 and 1284 were further subdivided into additional MLVA-types, as were ST38 and ST131 by adding the GECM-10 loci to those of GECM-7. Thus, MLVA generated substantial additional information to that of MLST.

Even though MLVA detected fewer types than PFGE, a substantial number of GECM-7-types were generated, and the PFGE clusters of isolates with similarities ≥80% generally corresponded to a single GECM-7 type. Also, in a few cases, GECM-7 analysis had a high discriminatory efficacy and distinguished isolates within a PFGE ≥ 80% similarity cluster as non-identical, suggesting that the two methods could complement each other, comparable to what has been described for PFGE and spa-typing for analysing MRSA [41].

ST131 is known to encompass a relatively wide range of PFGE-types, including O25b and its subclones [42]. As expected, the discriminatory power of MLVA for the various O25b-ST131 isolates was considerably lower than that of PFGE. Variation in the number of repeats was seen for this linage only within two MLVA loci, with the remaining eight loci being monomorphic. For the non-ST131 isolates, the variation within loci was considerably higher. However, MLVA clearly distinguished isolates of the ST131-O25b lineage from other isolates, including other ST131 isolates. It delineated these isolates further, as compared to PCR typing of O25b-ST131 and to the results obtained using the DiversiLab System or MALDI-TOF mass spectrometry [2, 43–45], and provided additional information of value for the investigation of the described outbreak. However, our results indicate that complementary methods are needed to verify or uncover strain relatedness within the O25b-ST131 lineage and its H30-Rx subclone. The majority of the O25b-ST131 isolates in this study belonged to the H30-Rx subclone. Even though this subclone is widespread and of growing importance clinically [2, 18], a different set of O25b- ST131 isolates might give different results, depending on the local diversity of the lineages and the extent of strain importation to the area.

For the time period studied and in our low-level endemic setting, it would have been sufficient to screen using the three loci of the GECM-3 protocol, to obtain rapid, reproducible and very easily interpreted typing information that could distinguish between isolate identity and non-identity in a majority of cases. Sorting out O25b-ST131 isolates beforehand by a PCR or more recently described methods, might prove to be a time-saving approach [44, 45]. Also, identity within epidemiologically related isolates needs to be confirmed or elucidated with additional loci or using additional methods for resolving the most similar types. Since the MLVA method is based on the analysis of loci with a high degree of genetic variation [2, 25, 36, 46], the MLVA patterns of strains disseminated within a specific locale are likely to vary over time. Therefore, if the GECM-3 protocol is used routinely, it is important to ascertain on a regular basis that the loci chosen are able to discriminate between the isolates that are circulating at the time.

Experience using MLVA for the typing of ESBL-*E.coli* remains limited, with few published studies [22, 36, 38, 40, 47, 48], and the lack of a common database for researchers is a disadvantage. WGS is being applied more and more in typing, especially for outbreak investigations [19]. Despite dramatic technological progress in recent years, WGS is not yet readily applicable to epidemiological surveillance of larger numbers of isolates for the everyday work of many clinical laboratories, especially with regard to assembly and interpretation issues [49]. On the other hand, with an improving capability of WGS to detect repetitive units, applying the MLVA concept and identifying distinct variable parts of the genome and comparing these sequences, without the need to interpret the whole genome, may indeed be a way to apply WGS techniques with a MLVA approach in the future, thereby also enabling the exchange of MLVA-like data between laboratories.

## Conclusion

With the alarming reports that the frequency of carbapenemase-expressing *Enterobacteriaceae* is increasing worldwide [50], also in low-level endemic settings such as Scandinavia [51], the surveillance of multidrug-resistant *Enterobacteriaceae* becomes increasingly important. Variants of MLVA, including a method reported for analysing *Klebsiella* spp [52], could play important roles as an effective first-step “screening” protocol to exclude clonal relationships between isolates and to decide whether an outbreak investigation should be initiated, since prompt action is crucial in preventing further transmission. We believe that the rapidity, technical simplicity and ease of interpretation of MLVA, not least the mini-GECM approach, makes it suitable as a “first-line-of-defence” method in the epidemiological surveillance of multidrug-resistant *E. coli,* preferably after first sorting out isolates that are part of the O25b-ST131 lineage. It may also act as a useful complement to traditional typing methods, particularly at the local level, not the least for smaller diagnostic laboratories. Indeed, the concept of MLVA may prove to be even more useful when WGS technologies become more readily available to most laboratories.

## Abbreviations

ESBL: Extended-spectrum beta-lactamases
GECM: Generic *E. coli* MLVA
GECM-10: GECM using ten loci
GECM-3: GECM using three loci
GECM-7: GECM using seven loci
H30-Rx ST131: FimH30 lineage, subclone of ST131
MLST: Multi locus sequence typing
MLVA: Multiple-locus variable number tandem repeat analysis
PFGE: Pulsed-field gel electrophoresis
ST: Sequence type
VNTR: Variable number of tandem repeats
WGS: Whole genome sequencing
